# Bi-Specific Killer Cell Engager Enhances NK Cell Activity against Interleukin-13 Receptor Alpha-2 Positive Gliomas

**DOI:** 10.3390/cells12131716

**Published:** 2023-06-25

**Authors:** Kristen D. Pawlowski, Joseph T. Duffy, Arushi Tiwari, Markella Zannikou, Irina V. Balyasnikova

**Affiliations:** 1 Department of Neurological Surgery, Northwestern University, Chicago, IL 60611, USA; 2 Rush Medical College, Rush University Medical Center, Chicago, IL 60612, USA

**Keywords:** glioblastoma, natural killer cells, bi-specific antibody, immunotherapy

## Abstract

Glioblastoma (GBM) is a lethal brain tumor with limited therapeutic options. Bi-specific killer cell engagers (BiKEs) are novel immunotherapies designed to engage natural killer (NK) cells against cancer. We designed a BiKE molecule consisting of a single-domain CD16 antibody, an interleukin-15 linker, and a single-chain variable antibody against the glioma-associated antigen interleukin 13 receptor alpha 2 (IL13Rα2). Recombinant BiKE protein was expressed in HEK cells and purified. Flow cytometric analysis of co-cultures of peripheral blood-derived NK cells with GBM6 and GBM39 patient-derived xenograft lines revealed significantly increased activation of NK cells (CD25+CD69+) and increased glioma cell killing following BiKE treatment compared to controls (n = 4, *p* < 0.01). Glioma cell killing was also confirmed via immunofluorescence staining for cleaved caspase-3 (*p* < 0.05). In vivo, intracranial delivery of NK cells with BiKE extended median survival in mice bearing GBM6 (*p* < 0.01) and GBM12 (*p* < 0.01) tumors compared to controls. Finally, histological analysis of brain tissues revealed a higher frequency of peritumoral NK cells in mice treated with BiKE than with NK cells alone (*p* < 0.05). In conclusion, we demonstrate that a BiKE generated in a mammalian expression system is functional in augmenting NK cell targeting of IL13Rα2-positive gliomas.

## 1. Introduction

Glioblastoma (GBM) is the most aggressive brain tumor in adults [1], with a median overall survival of less than 15 months [1,2]. The current standard of care consists of surgical resection followed by radiotherapy and chemotherapy using the alkylating agent temozolomide [3,4]. More recently, tumor-treating fields (TTFields), low-intensity alternating electric fields, has been introduced to the current standard of care, prolonging overall survival by 4 months [2]. Despite this, the prognosis for patients with GBM remains poor. Tumor recurrence is inevitable, and the five-year survival rate after diagnosis is approximately 6.8% [1,5]. Thus, there is a dire need for novel approaches to improve patient outcomes for this disease.

Immunotherapies are an attractive approach for the treatment of many cancers. Despite the promise they have shown in hematopoietic and extracranial malignancies, efficacy has yet to be shown in GBM [6]. The underlying resistance to immunotherapy in GBM is complex and undeniably multifactorial. The myeloid cell-dominated GBM tumor microenvironment (TME) is highly immunosuppressive and heterogeneous. The presence of glioma stem-like cells (GSCs), extremely infiltrative and resistant neoplastic cells, drive tumor recurrence and disease progression [7,8,9,10,11,12]. Several studies have examined the use of immune checkpoint inhibitors to restore and promote T cell function [13,14] and vaccine-based approaches aiming to prime T cells for glioma antigens [14,15,16], although neither has achieved clinical benefit. Recent efforts have focused on Chimeric Antigen Receptor (CAR) cell therapy, a type of engineered cell-based therapy that modifies T cells to target glioblastoma-specific antigens, such as interleukin 13 receptor alpha 2 (IL13Rα2), epidermal growth factor receptor variant III (EGRFvIII), human epidermal growth factor receptor 2 (HER2), and B7-H3 [17,18,19]. Despite showing promising anti-tumor activity in preclinical studies, high heterogeneity, limited persistence, and dampened proliferation in the highly immunosuppressive TME of patients thwarted clinical response [20,21,22].

Therapies targeting natural killer (NK) cells have grown in popularity in recent years. NK cells are cytotoxic lymphocytes that play a significant role in immune surveillance, exist independent of human leukocyte antigen (HLA) compatibility, have a low risk of graft-versus-host disease, and are naturally involved in the surveillance of cellular dysplasia [23]. They function primarily through the surface receptor CD16 and operate through antibody-dependent cell-mediated cytotoxicity (ADCC). Interaction of CD16 with the Fc portion of antibodies induces the release of perforin, granzymes, and cytokines (i.e., type I interferons and tumor necrosis factor) to activate apoptotic receptors in target cells [24,25,26,27,28]. Notably, this process occurs independently of antigen priming [24,29]. While NK cells have been shown to be present in the TME [23], most exist in an immunosuppressed, non-cytolytic state [12]. The NK cells that are active, however, have been shown to be effective in killing the GSCs that are responsible for tumor infiltration, therapy resistance, and eventual reoccurrence [30,31]. Since NK cells are a promising target for GBM, recent efforts to understand the mechanisms behind NK cell resistance have been an active area of study. For example, Shah et al. recently discovered an innate anti-glioma NK-mediated pathway under the control of galectin-1 (Gal-1) [32]. Gal-1 is a member of a family of β-galactoside-binding lectins and is used by glioblastoma tumors to suppress NK cell function [33]. Shah et al. demonstrated that the knockdown of Gal-1 in gliomas triggers the release of miR-1983 within exosomes, activating toll-like receptor 7 (TLR7) in DCs [32]. The activated DCs subsequently release IFN-β, which stimulates NK cells to kill glioma tumors via Perforin and Granzyme B. However, in wild-type glioblastoma, the tumors express Gal-1 allow evasion from NK cells. Thus, developing therapeutic strategies to overcome NK cell suppression by glioma is of particular interest.

Bispecific engagers consist of antibodies that directly link immune cells with target cells to facilitate an effector response [34]. In immuno-oncology, these engineered molecules typically consist of an antibody against a tumor-specific antigen, a peptide linker, and another antibody that recognizes an effector cell [35]; in the case of bi-specific killer cell engagers (BiKEs), the effector antibody recognizes an antigen on NK cells. Multiple studies have examined BiKE efficacy in hematological (i.e., acute myeloid leukemia (AML) [36]) and solid (i.e., colorectal [34], lung [37]) malignancies. However, their use in GBM has yet to be explored. In this study, we engineered a BiKE equipped with a single-domain antibody against CD16, a stimulatory molecule on NK cells, and a single-chain antibody against IL13Rα2, an antigen overexpressed in 60% of glioblastoma tumors [38,39]. Notably, IL13Rα2 is not expressed in healthy brain tissue [38] and is associated with glioma migration and invasion [39], making it an especially attractive antigen to target GBM cells. Finally, to potentiate NK effector function, we incorporated an interleukin-15 (IL-15) linker, a pro-inflammatory cytokine with potent effects on NK cell survival, growth, and proliferation [40,41]. The effect of BiKE on NK cell activation was investigated using GBM spheroids in vitro and patient-derived GBM xenografts in mice.

## 2. Materials and Methods

### 2.1. Construction of BiKE

Synthesis of assembled hybrid genes encoding BiKE was done by GenScript (Piscataway, NJ, USA). BiKE consists of four parts: (i) a VH region of human CD16 (GenBank EF561291.1) as a 20 amino acid segment (PSGQAGAAASESLFSNHAY) [42] originally generated by Behar et al., [43], (ii) a modified interleukin-15 (IL-15) (GenBank U14407.1) as a 7 amino acid segment (EASGGPE) [42], (iii) a human VH and VL region of human interleukin 13 receptor alpha 2 (IL13Rα2) [44], and (iv) a 6HIS tag incorporated for purification and detection. A separate single-domain CD16 (sdCD16) antibody was generated as a control and consisted of (i) the VH region of human CD16 and 6HIS tag. Final sequences were validated by GenScript. Complementary DNA encoding BiKE and sdCD16 were subcloned in EcoRI and BamHI restriction sites of pLVX-IRES-zsGreen1 lenti viral vector (Takara Bio USA, San Jose, CA, USA), transfected and packaged into lentiviral particles (Lenti-X Packaging Single Shots, Takara Bio USA), and subsequently transduced into HEK 293 T/17 cells (ATCC Scientific, North Little Rock, AR, USA) and enriched via fluorescent activated cell sorting. The estimated molecular weight of BiKE and CD16 Nb (sdCD16) have was 59.3 and 16.26 kDa, respectively (https://www.bioinformatics.org/sms/prot_mw.html, accessed on 14 April 2020).

### 2.2. Optimization of BiKE Production

We performed a series of optimization experiments to ensure optimal production of soluble BiKE. Optimization consisted of both temperature and serum conditions for cell culture of 293 T/17 cells secreting BiKE. BiKE production under two temperatures, 32 °C and 37 °C, was evaluated in BiKE-producing HEK 293 cells grown in DMEM media (Corning, Manassa, VA, USA), and the production under three serum conditions, DMEM with 10% fetal bovine serum (FBS) (Atlanta Biologicals, Flowery Branch, GA, USA), DMEM with 1% FBS, and OptiMEM^TM^ media (ThermoFisher Scientific, Waltham, MA, USA), was evaluated [45]. All media was supplemented with 1% penicillin/streptomycin (Corning). Western blotting was used to visualize the BiKE production in each condition. Briefly, lysates were prepared by collecting the supernatant and mixing in 4× Laemmli sample buffer (Bio-Rad, Hercules, CA, USA). A non-diluted (ND) sample and a 2× diluted sample (2×) were prepared for each set of temperature or serum conditions. Samples were diluted in purified water, heated at 95 °C for 10 min, and separated via electrophoresis using a 10% SDS–polyacrylamide gel. Separated proteins were transferred to a PVDF membrane, blocked for 1 h in 5% non-fat dry milk, and incubated for 1 h at room temperature with anti-6X His tag HRP-conjugated antibody (Abcam, Cambridge, MA, USA) (1:5000 in 5% non-fat milk). The membrane was then washed with 0.05% Tween 20 in PBS (PBST), incubated with Clarity Western ECL Substrate (Catalog number 1705061; Bio-Rad), and imaged with the Bio-Rad ChemiDoc Imaging System.

### 2.3. Production and Purification of BiKE

The 293T/17 cells secreting BiKE or sdCD16 were expanded at 37 °C, and then transferred to a 32 °C incubator for 2–3 days in the presence of protease inhibitors (Sigma-Aldrich, St. Louis, MO, USA) to maximize protein production. Supernatants from the 293T/17 cells containing recombinant proteins were collected and subsequently purified using cOmplete^TM^ His-Tag Purification Resin (Roche, Basel, Switzerland). The resin was incubated with supernatants overnight at 4 °C on a rotating shaker. The resin was transferred in cartridges and washed with at least 20 column volumes of buffer A (50 mM Tris-HCl, 1 M NaCl, pH 8.0). Protein was eluted with buffer B (50 mM Tris-HCl, 1 M NaCl, 250 mM imidazole, pH 8.0). Eluted fractions were monitored using a Bradford Coomassie Blue Reagent (Cat 1856209, Lot WA314734; Thermo Scientific, Rockford, IL, USA). Fractions with the greatest signal were pooled and dialyzed using 1× PBS overnight at 4 °C. Protein was then concentrated using an Amicon Ultra-0.5 Centrifugal Filter Unit (10 kDa cutoff) (Millipore Sigma, Burlington, MA, USA). The protein concentration was measured using the NanoDrop (Thermo Scientific) and stored at −80 °C until use.

### 2.4. Glioma Cell Lines and Cell Culture

Human patient-derived glioma cell lines GBM6, GBM12, and GBM39 were propagated in mouse flanks before use in culture [46]. GBM6 and GBM39 spheroids were generated in human neurobasal media (human Neurocult NS-A Basal Medium; Cat 5750; StemCell Technologies, Vancouver, Canada) supplemented with 20 ng/mL human recombinant EGF (Cat 78006; StemCell Technologies), 10 ng/mL human recombinant beta-FGF (Cat 78003; StemCell Technologies), 0.5% StemPro Neural Supplement proliferation media [Cat A1050801; Thermofisher Scientific), and 1% penicillin/streptomycin). Cells were grown in a humidified incubator at 37 °C and 5% CO_2_.

### 2.5. Human PBMC-Derived NK Cell Isolation

Fresh, healthy donor blood was collected into a BD Vacutainer supplemented with ethylenediamine tetraacetic acid (EDTA) (Cat 367841; BD Biosciences, Haryana, India) and processed within 1 h of collection. Whole blood was diluted with PBS at a 1:1 volume ratio and layered at a 4:3 ratio onto Ficoll-Paque PLUS density gradient (Cat 17144003; Cytiva, Marlborough, MA, USA). Layers were obtained via centrifugation at 400× *g* for 45 min without brake. NK cells were subsequently isolated from peripheral blood mononuclear cells (PBMCs) using immunomagnetic negative selection (MojoSort Human NK Cell Isolation Kit, Cat 480053, Lot B359206; BioLegend, San Diego, CA, USA) and used immediately for in vitro experiments.

### 2.6. Human NK Cell Expansion

PBMCs were obtained as previously described from fresh human blood and expanded ex vivo using a commercially available kit (CellXVivo Human NK Cell Expansion Kit, Cat CDK015a, Lot P309494; R&D Systems, Inc., Minneapolis, MN, USA) per manufacturer’s protocol.

### 2.7. BiKE Binding Studies and Quantification

Enzyme-linked immunosorbent (ELISA) assay was used to determine the binding capacity of BiKE to human recombinant IL13Rα2 (Cat 7147-IR-100; R&D Systems). A 96-well plate was coated overnight at 4 °C with 1 μg/mL IL13Rα2 in PBS. The plate was washed three times with PBST, blocked with FACS buffer (PBS, 1% BSA) for 2 h at room temperature (RT), and washed five times with PBST. Purified BiKE proteins were incubated at various concentrations and dilutions for 2 h at RT, and the plate was washed six times with PBST. Bound proteins were detected with HRP-conjugated anti-6X His tag antibodies (Cat ab1187; Abcam), visualized using 1-Step^TM^ Slow TMB-ELISA (Cat 34024; Thermo Scientific) and neutralized 2N HCl. Plates were read at 450 nm using a BioTek plate reader (BioTek, Winooski, VT, USA).

### 2.8. Flow Cytometry

PBMC-derived purified NK cells were incubated for 24 h at 37 °C, 5% CO_2_ in RPMI supplemented with 5% AB human serum (Cat HS-20; Omega Scientific, Tarzana, CA, USA), and 1% PS with GBM6 or GBM39 spheroids and treated with either 10 μg/mL of BiKE or sdCD16. Cells were centrifuged at 350 RCF for 10 min at 4 °C and blocked with TruStain FcX anti-mouse CD16/32 (Clone 93, Cat 101319; BioLegend) at a 1:100 in FACS buffer for 20 min at 4 °C. Cells were subsequently stained with the following monoclonal antibodies at a 1:50 dilution in FACS buffer for 30 min at 4 °C in the dark: APC-conjugated anti-human CD16 (Clone 3G8, Cat 302011; BioLegend), PE/Cy7-conjugated anti-human CD56 (Clone HCD56, Cat 318318; BioLegend), BUV 395-conjugated anti-human CD45 (Clone HI30, Cat 563792; BD Horizon, Franklin Lakes, NJ, USA), PE-conjugated anti-human CD25 (Clone BC96, Cat 302606; BioLegend), FITC-conjugated anti-human CD69 (Clone FN50, Cat 310903; BioLegend), and BV 711-conjugated NKp46 (Clone 9E2, Cat 331935; BioLegend) in FACS buffer for 30 min at 4 °C in the dark. Cells were then stained with live/dead fixable viability stain APC/Cy7 (Cat 65-0865-14, Lot 2365395; Invitrogen, Waltham, MA, USA) at 1:1000 in PBS for 15 min at 4 °C in the dark. Cells were fixed in 1% PFA and analyzed on the BD Fortessa flow cytometer (BD Horizon) at the Robert H. Lurie Comprehensive Cancer Center Flow Cytometry Core Facility. Flow cytometric analyses were performed in FlowJo software (v10.9.0, FlowJo, LLC, Ashland, OR, USA).

### 2.9. Caspase-3 Assay

Co-culture experiments of PBMC-derived NK cells and GBM6 spheroids were performed as previously described. After 24 h of treatment with BiKE, cells were spun down at 350 RCF for 5 min, resuspended in 4% PFA, and left at room temperature for 15 min. Cells were washed thrice with 1% bovine serum albumin (BSA)/PBST (PBS with 0.3% Tween 20). Cells were permeabilized with 0.1% triton X-100 (Lot SLCJ6163; Sigma-Aldrich) in PBS for 10 min at RT. Cells were washed thrice with PBST and blocked with 5% BSA/0.3% triton X-100/PBS at RT for 60 min. Cells were washed three times and incubated with Caspase-3 antibody (Rabbit pAb Caspase-3, ab4051, lot GR3359534-3; Abcam) (1:250 dilution in 1% BSA/0.3% triton X-100/PBS) overnight in the dark at 4 °C. Cells were washed five times and incubated with secondary antibody (Alexa Fluor 647 goat anti-rabbit IgG H+L], A-21244, lot 2390713; Invitrogen) (1:1000 dilution in 1% BSA/0.3% triton X-100/PBS) at RT for two hours. Cells were washed three times with PBST, transferred into cytospin funnels (EZ Double Cytofunnel, Cat A78710005, Lot 22035A0; Epredia, Kalamazoo, MI, USA), and spun at 250 RCF for 2 min (Thermo Scientific Cytospin 4). Slides were mounted with ProLong Gold Antifade with DAPI (Cat P36931, Lot 2478336; Invitrogen), and fluorescence was read with a Leica DMi8 microscope (Leica Microsystems, Wetzlar, Germany). Subsequent analysis of images was performed using ImageJ software (version 1.53t, National Institutes of Health, Bethesda, MD, USA). Spheres were included in quantification if greater than 100 μm, did not appear flat, and had a strong distribution of DAPI. DAPI blue fluorescence was adjusted for a minimum of 15 and a maximum of 252. Caspase-3 red fluorescence was adjusted for a minimum of 7 and a maximum of 71. Corrected total cell fluorescence (CTCF) was calculated by subtracting the total area of the sphere (assessed via DAPI-staining) multiplied by the mean fluorescence of background readings from the integrated density.

### 2.10. Survival Analysis

All animal research was conducted in accordance with the Northwestern University Institutional Animal Care and Use Committee (IACUC) guidelines (protocol IS00002999). Six- to eight-week-old B6.129S7-Rag1^tm1Mom^IJ (Jackson Laboratory, Bar Harbor, ME, USA) were used for intracranial implantation of human GBM6 or GBM12 glioma xenografts. The mice were anesthetized with ketamine/xylazine anesthesia, and 2.0 × 10^5^ GBM6 or GBM12 cells were delivered stereotaxically to each mouse as previously described [47]. Expanded human NK cells (1 × 10^6^ cells per mouse) were injected at the same location 7 days post-tumor implantation with or without 10 μg of BiKE, sdCD16, or saline.

### 2.11. Immunohistochemistry

Tumor tissues were collected one to three days following NK cell treatment in mice. Briefly, mice were anesthetized with a ketamine/xylazine and perfused with PBS via the left ventricle until the liver and lungs were cleared of blood. The brain was carefully excised, placed in 10% formalin, and stored at 4 °C. PBS was exchanged between 24 h after fixation. Tissue antigens were retrieved via heat-induced epitope retrieval using sodium citrate buffer (10 mM sodium citrate, 0.05% Tween 20, pH 6) at a maximal temperature of 110 °C and prepared in 4 μm sections. Since human NK cells were the only lymphocyte introduced into mice, NK cells were stained using a monoclonal antibody specific to human CD45 (clone 2B11 & PD7/26, cat 145M-98, undiluted; Sigma-Aldrich), which was detected using the Streptavidin–Biotin complex method, as previously described [48]. Tumors were alternatively stained with the polyclonal antibody to cleaved caspase 3 (Cat ab4051, 1:100 dilution; Abcam), which was detected using a polymeric HRP-conjugated anti-IgG antibody (DAB Polymer Refine Detection Kit, Cat DS9800; Leica Microsystems) to quantify tumor cell apoptosis. Slides were counterstained with hematoxylin and embedded in mounting medium. Tumor size was calculated using ImageJ. Quantification of CD45-positive or caspase-positive cells within the peritumoral area were calculated for three tumor slices per animal and averaged. Three animals were included in each treatment group, and group averages were compared in statistical analysis. Representative images were prepared for publication using NDP.view2 software (U12388-01, Hamamatsu Photonics, Shizuoka, Japan).

### 2.12. Statistical Analysis

Data analysis was performed using R (R Core Team 2021, Indianapolis, IN, USA) [49]. A p-value of 0.5 was determined to be statistically significant in all studies. An Independent two-sample t-test was used to compare between two means. One-way ANOVA was used for multiple comparisons. Animal survival was assessed via the Kaplan-Meier curve using the log-rank method to determine the *p*-value. All data are presented as an average of at least 3 replicates, and error bars represent the standard error of the mean.

## 3. Results

### 3.1. Optimization of BiKE Production in a Mammalian Expression System

**Figure 1:**
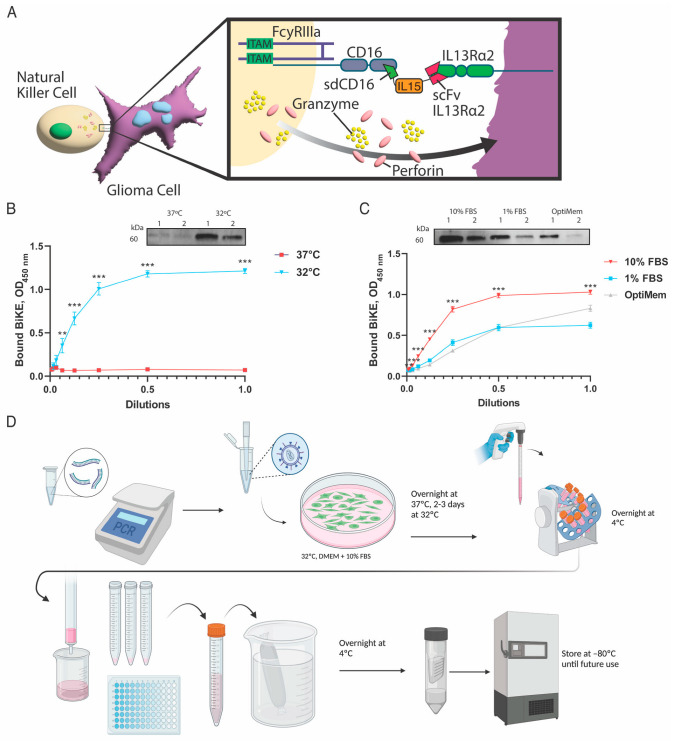
Optimization of BiKE production. (**A**) Schematic drawing representing BiKE mediated engagement of NK cells and IL13Rα2-positive glioma cells. The construct consists of an anti-CD16 single-domain antibody (sdCD16), interleukin-15 flanked by flexible protein linkers (IL15), an anti-IL13Rα2 single-chain fragment antibody (scFv IL13Rα2). (**B**) Optimization of BiKE production regarding the production temperature was conducted at 37 °C and 32 °C. Visualization of protein yields was conducted using Western blot, and binding to IL13Rα2 was demonstrated after production at 32 °C. (**C**) BiKE optimization for serum conditions was conducted in DMEM media with 10% FBS (10% FBS), DMEM with 1% FBS (1% FBS), and OptiMEM media. BiKE demonstrated the greatest production and maximal binding capacity to its target using the 10% FBS condition. (**D**) Schematic overview of BiKE production. Production can be efficiently completed in 6-7 days. First, BiKE cDNA was inserted into a pLVX-IRES-zsGreen1 lentiviral vector. A stable 293 T/17 cell line secreting BiKE was generated through lentivirus transfection. 293 T/17 cells producing cells were incubated overnight at 37 °C and transferred to 32 °C for another 2 to 3 days. The supernatant containing BiKE was collected, incubated with cOmplete His-Tag resin overnight at 4 °C, and isolated through His-Tag column purification. Purified BiKE was then dialyzed overnight with PBS serum, concentrated, and stored at −80 °C for future use. ** = *p* < 0.01, and *** = *p* < 0.001.

The BiKE used in this work consists of a VH region of human CD16, a modified interleukin 15 (IL-15) flanked by two flexible protein linkers [42], a human VH and VL region of human interleukin 13 receptor alpha 2 (IL13Rα2) antibody (clone 47) [44], and 6HIS tag (Figure 1A).

A single-domain CD16 nanobody (sdCD16) was generated as the control. BiKE production in 293T/17 cells was optimized for temperature and serum conditions. First, producer cells were grown at either 32 °C or 37 °C in DMEM media supplemented with 10% FBS. Western blot demonstrated increased protein production at 32 °C (Figure 1B). The binding of purified BiKE to coated IL13Rα2 protein was assessed by ELISA and showed that the BiKE had optimal production yields after culturing at 32 °C as compared to 37 °C (*p* < 0.001) (Figure 1B). Next, serum conditions of DMEM with 10% FBS (10% FBS), DMEM with 1% FBS (1% FBS), and OptiMEM media were tested. BiKE showed maximal yield and hence maximal binding in ELISA when produced in DMEM supplemented with 10% FBS (Figure 1C). Overall, BiKE production can be efficiently completed in 6 to 7 days (Figure 1D).

### 3.2. BiKE Activates NK Cells to Augment Glioma Cell Killing In Vitro

**Figure 2:**
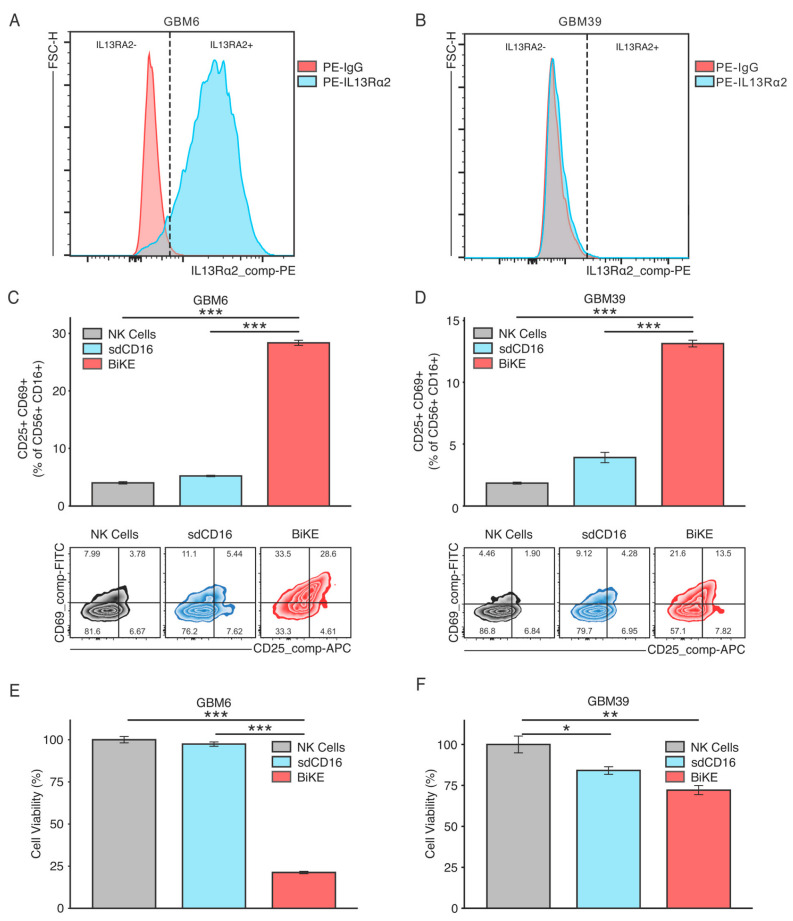
BiKE activates PBMC-derived NK cells towards IL13Rα2-positive gliomas. GBM6 or GBM39 spheroids were co-cultured for 24 h with either NK cells alone (NK Cells) or in combination with sdCD16 (sdCD16) or BiKE (BiKE). Expression levels of IL13Rα2 in both (**A**) GBM6 and (**B**) GBM39 are provided. BiKE significantly increased NK activation markers CD25 and CD69 in both (**C**) GBM6 and (**D**) GBM39 co-culture conditions. Treatment with BiKE resulted in significantly more killing of tumor cells than sdCD16-treated NK cells, NK cells alone, or controls in both (**E**) GBM6 and (**F**) GBM39 co-culture. * = *p* < 0.05, ** = *p* < 0.01, and *** = *p* < 0.001.

BiKE-mediated activation of NK cells was assessed in co-culture with two established patient-derived glioma xenograft lines, GBM6 and GBM39. GBM6 is a high IL13Rα2 expressing GBM cell line (Figure 2A); GBM39 is a low IL13Rα2-expressing GBM cell line (Figure 2B). Therefore, the selection of these two cell lines permitted the testing of BiKE’s sensitivity to its target, IL13Rα2.

**Figure 3:**
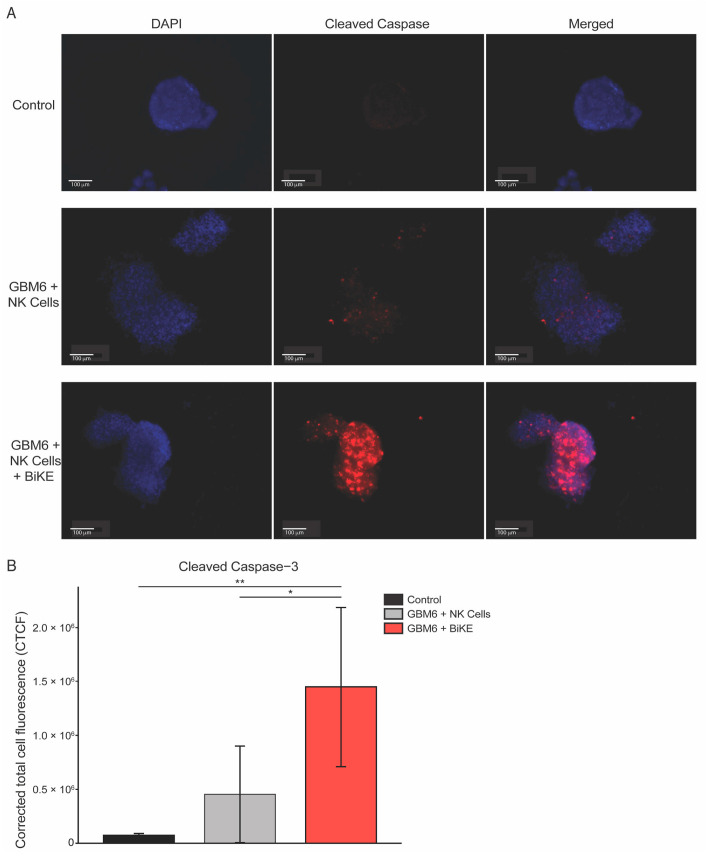
BiKE treatment in GBM6 spheroids increased tumor cell killing via immunofluorescent detection of cleaved caspase-3 staining with PBMC-derived NK cells. GBM6 spheroids were co-cultured for 24 h with either PBMC-derived NK cells alone or treated with 10 μg of BiKE. (**A**) Representative images from left to the right demonstrate markers for DAPI shown in blue, cleaved caspase-3 shown in red, and respectfully merged DAPI and cleaved caspase-3. (**B**) Quantification of cleaved caspase-3 demonstrates a significant increase in GBM6 spheroid killing with BiKE-treated NK cells compared to NK cells alone or GBM6 spheroids alone. * = *p* < 0.05 and ** = *p* < 0.01.

Glioma cells were co-cultured either with PBMC-derived NK cells alone (NK Cells), NK cells with sdCD16 (sdCD16), or NK cells with BiKE (BiKE) for 24 h. NK activation was assessed via flow cytometric analysis of activation markers CD25 and CD69. BiKE significantly increases cell surface expression CD25 and CD69 in both GBM6 (Figure 2C) and GBM39 (Figure 2D) co-cultures, albeit at a lower amount in GBM39 as to be expected by its lower expression level of the receptor. The killing of glioma cells was also assessed through flow cytometry. BiKE showed the greatest amount of tumor killing in the GBM6 cell line (Figure 2E) versus GBM39 (Figure 2F), redemonstrating that BiKE has the most effective response when the receptor is highly expressed. Finally, as BiKE is believed to function through the cytotoxic action of NK cells, the evaluation of GBM6 tumor cell death was also evaluated via cleaved caspase-3 immunofluorescence. As expected, a significant increase in caspase activation was seen in BiKE-treated co-cultures compared to controls (Figure 3A,B).

Overall, BiKE can activate NK cells and induce the killing of glioma cells with various expression levels of the target IL13Rα2.

### 3.3. BiKE Prolongs Survival of GBM6 and GBM12 Tumor Bearing Mice

**Figure 4:**
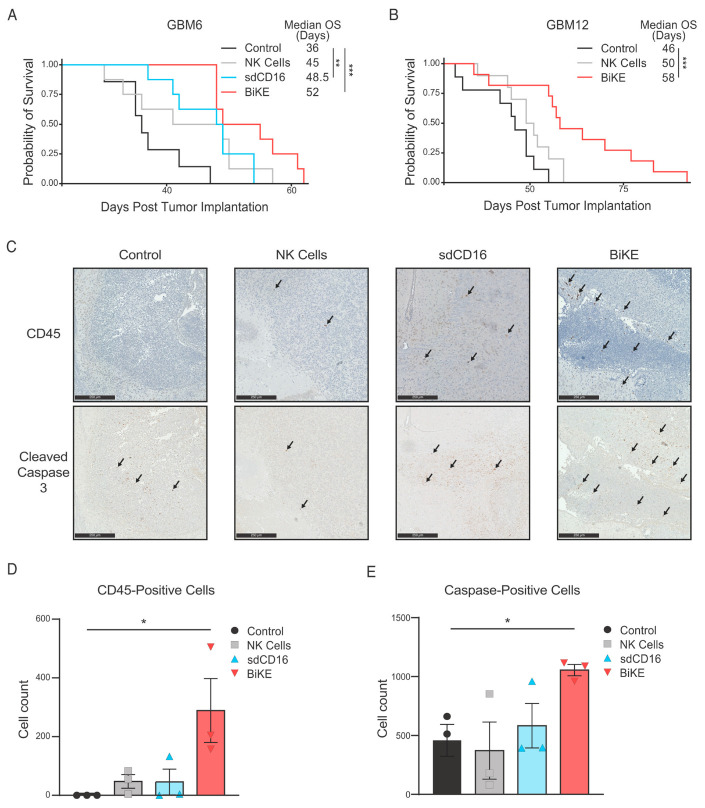
BiKE extends survival of mice bearing patient-derived glioma xenografts through augmenting NK cell presence intratumorally and increasing tumor cell death. (**A**) GBM-6 xenografted animals treated with BiKE survived significantly longer than those treated with expanded NK cells alone or saline (Kaplan-Meier survival curves were compared using log-rank tests). (**B**) GBM12-xenografted animals demonstrated similar results. (**C**) Immunohistochemistry images with stain for NK cells (CD45-positive lymphocytes) and dead tumor cells (cleaved caspase-3, indicated by arrows) are shown for GBM6 mice sacrificed 1–3 days following treatment. Images shown are at 10× magnification. The scale bar is equal to 250 µm. Quantification of (**D**) CD45-positive lymphocytes and (**E**) cleaved caspase-3 positive cells are provided. Three brain slices were analyzed for each animal, and positive cells were averaged. The final cell count per treatment group is represented by the average of these individual values (n = 3). * = *p* < 0.05, ** = *p* < 0.01, and *** = *p* < 0.001.

Since the BiKE molecule showed the greatest glioma killing in vitro with high receptor expression, we sought to test survival benefits in two xenograft models with high-expression levels of IL13Rα2, both GBM6 and GBM12 [47]. Seven days post tumor engraftment, tumor-bearing mice were injected intracranially with either PBS (Control), NK cells, NK cells with sdCD16 (NK Cells + sdCD16), or NK cells with BiKE molecule (NK Cells + BiKE). Treatment with BiKE is significantly prolonged survival compared to controls of mice bearing GBM6 tumors, with a median survival of 52 days compared to 36 days, respectively (Figure 4A, *p* < 0.001).

sdCD16 molecule additionally prolonged survival in comparison to controls to 48.5 days, albeit to a lesser degree than BiKE (Figure 4A). BiKE efficacy was tested in a second GBM PDX model, GBM12. BiKE treatment significantly improved the survival of GBM12-bearing mice, with a median survival of 58 days in the BiKE-treated group versus 46 days within control animals (Figure 4B). A separate cohort of twelve mice bearing GBM6 xenografted mice were sacrificed one to three days following treatment with BiTE. The brains underwent immunohistochemical (IHC) analysis to analyze NK cell presence and tumor cell killing. Analysis of human CD45+ cells revealed significantly more NK cells in the tumor of BiKE-treated mice compared to both controls and NK or sdCD16 treatment groups (Figure 4C,D, *p* < 0.05). Similarly, analysis of cleaved caspase 3 revealed significantly more apoptotic cells in BiKE-treated animals compared to both NK cells alone and controls (Figure 4C,E). Taken collectively, BiKE promotes the persistence of NK cells within the tumor site, increases tumor cell death, and extends survival in GBM-bearing animals.

## 4. Discussion

This study reports the design, generation, and functional analysis of therapeutic BiKE targeting for IL13Ra2-positive gliomas. The BiKE, consisting of a single-domain anti-CD16 antibody, an IL-15 linker, and an scFv anti-IL13Ra2, can be reliably manufactured in a mammalian expression system. BiKE facilitates the engagement of NK cells and glioma cells, directs NK cell-mediated cytotoxicity of IL13Ra2-positive gliomas, and extends the survival of GBM-bearing animals.

The success of any targeted immunotherapeutic depends on its on-target and on-tumor capability. Outside of the testis, IL13Ra2 is expressed at low levels in the body and is not expressed in a normal brain [38]. However, IL13Ra2 is overexpressed in 60% of glioblastoma tumors [38,50,51]. This makes IL13Ra2 an especially attractive antigen to target GBM cells and mitigate the on-target off-tumor toxicity of targeted immunotherapeutics. Our lab previously developed an scFv antibody that targets IL13Ra2 that has been successfully incorporated into multiple CAR T-cell [44,47,52,53] and bi-specific T-cell engager (BiTE) therapies [47]. Here, we described a BiKE molecule that successfully targets IL13Ra2-positive gliomas even with low receptor expression, albeit at a lesser degree than high-expressing IL13Ra2-positive gliomas. These data warrant studies of BiKE and NK cells in GBM models recapitulating a heterogenous expression of IL13Ra2 in patients’ GBM tissue.

NK effector activity is modulated by the signaling summation from a multitude of surface receptors present in NK cells. Signaling following ligand engagement of natural cytotoxicity receptors (NCRs), such as NKp30 and NKp46, C-type lectin superfamily receptors, such as NKG2D, and signaling lymphocytic activation molecules, such as DNAM-1, all modulate NK cell cytolytic activity [54]. However, ADCC in NK cells relies on interactions through Fc receptors, and BiKE directs NK cell-mediated cytotoxicity through engagement with the FcγRIII (e.g., CD16) [54]. BiKE incorporated an sdCD16, and our results showed increased expression of CD69 and CD25 activation markers in NK cells following treatment with both BiKE and sdCD16. Similarly, numerous other studies have also demonstrated that when sdCD16 is incorporated into a BiKE, it activates NK cells and directs NK cell-mediated cytotoxicity of tumor cells [55,56,57,58,59,60]. However, a growing body of evidence describes split anergy when NK cells are treated with an F(ab′)2 fragment of an anti-CD16 antibody. This split anergy is characterized by reduced activation, reduced cytolytic activity, increased IFNg production, reduction in mature cathepsins C and H, and promotion of NK inhibitory enzyme cystatin F [61,62,63,64,65]. Many differences exist between the anti-CD16 F(ab′)2 used in studies describing NK cell split anergy and sdCD16 used in BiKE. F(ab′)2 molecules are 7–8 times larger than single-domain antibodies [66]. Although two antibodies were not directly compared in our studies, it is tempting to speculate that the binding of larger divalent F(ab)2 and smaller monovalent sdCD16 antibodies, along with their different epitopes, might result in distinct conformational changes in CD16 [67]. Lastly, we only analyzed activation of NK cells at a 24-h timepoint, whereas other studies describing split anergy used a 48–72 h timepoint. Thus, future studies are needed to better understand the differences between sdCD16 and anti-CD16 F(ab′)2 regarding split anergy.

While NK cells are known to be present in the GBM TME, the numbers of NK cells existing within the tumor site can vary considerably [23], with some studies reporting insignificant NK cell infiltration [68,69] and other studies showing NK cell presence at 26% of all infiltrating lymphocytes. A study by Zhu et al. suggests that the risk group of the tumor may be correlated with NK cell infiltration, with high-risk tumors having a larger infiltrate [70]. Ultimately, the effectiveness of any cell-based therapy depends on the survival, persistence, and maintenance of effector cell populations in the TME. To this end, we included an IL-15 linker portion in our BiKE molecule to promote NK cell growth and function in and around the tumor site [43]. Our study shows an increase in NK cells within the GBM TME (Figure 4C,D) compared to sdCD16 or NK cells alone. However, our work is limited in assessing NK cell recruitment to the tumor site as we utilized a local injection of NK cells and BiKE protein instead of a systemic injection. Future studies investigating methods to increase NK cell recruitment into the TME to augment BiKE efficacy, such as combination therapy with chemokine therapy (i.e., CX3CL1 [71]), checkpoint blockade therapy (anti-PD1 treatment [72]), or drug delivery systems (i.e., nano-encapsulation and focused ultrasound [73,74]) are warranted.

Effector cells infiltrating GBM become functionally compromised in the immunosuppressive TME, and NK cells are no exception. Glioblastoma has been shown to downregulate classic MHC class I (MHC-I) molecules, the primary inhibitory molecule for NK cells [75]. It thus should increase NK-cell killing of the tumor. Despite this, NK cells still exist in an inhibited state intratumorally. In addition, recent evidence has shown that MR1, a nonclassical MHC class I-like molecule, is epigenetically overexpressed in glioma and correlated with poor overall survival and global dysregulation of many immune-related genes [76].

Transforming growth factor beta (TGF-β) is an immunosuppressive cytokine found at high levels in GBM [77,78,79] and can downregulate NK-activating receptors, such as NKG2D, to suppress NK cell effector function [77,78,79]. Futhermore, upregulation of other molecules by glioma cells, such as Gal-1, can facilitate evasion from the innate anti-glioma activity of NK cells [32]. Therefore, stimulating NK cells via the BiKE molecule’s binding to the CD16 receptor might overcome the inhibitory state of NK cells in the GBM TME. Finally, the unique ability of CD16 to activate NK cells without any additional receptor makes it a potent target for directing NK cell-mediated cytotoxicity. Our data showed the power of BiKE to induce activation of NK cells judged by the expression of CD25 and CD69 markers and mediate the killing of glioma cells in vitro and in vivo.

## 5. Conclusions

In this study, we demonstrate that BiKE can be generated in a mammalian expression system, and it is fully functional. Furthermore, our in vitro data highlight the ability of BiKE to activate NK cells, mediate the killing of IL13Rα2-positive tumors, and increase the survival of IL13Rα2-positive glioma-bearing animals. These results warrant further investigating BiKE as a treatment for IL13Rα2-positive glioblastoma.

## 6. Patents

I.V.B. has a patent for the use of ScFv47 for IL13Rα2-targeted cancer therapies. No other competing interests.

## Data Availability

All generated data are presented in the manuscript.
